# Impact of dry-off and lyophilized *Aloe arborescens* supplementation on plasma metabolome of dairy cows

**DOI:** 10.1038/s41598-023-31922-z

**Published:** 2023-03-31

**Authors:** L. Cattaneo, G. Rocchetti, F. Piccioli-Cappelli, S. Zini, E. Trevisi, A. Minuti

**Affiliations:** 1 Department of Animal Science, Food and Nutrition (DIANA), Faculty of Agricultural, Food and Environmental Sciences, Università Cattolica del Sacro Cuore, 29122 Piacenza, Italy; 2 Department of Molecular and Translational Medicine (DMMT), University of Brescia, 25121 Brescia, Italy; 3 Romeo and Enrica Invernizzi Research Center for Sustainable Dairy Production of the Università Cattolica del Sacro Cuore (CREI), 29122 Piacenza, Italy

**Keywords:** Metabolomics, Animal physiology

## Abstract

Positive effects have been observed as a result of *Aloe arborescens* supplementation in the dry-off phase in dairy cows. Metabolomic approaches can provide additional information about animal physiology. Thus, we characterized plasma metabolome around dry-off in 12 cows supplemented (AL) or not (CTR) with 10 g/d of lyophilized *A. arborescens* with an untargeted metabolomic approach. Overall, 1658 mass features were annotated. Regardless of treatment, multivariate statistics discriminated samples taken before and after dry-off. Overall, 490 metabolites were different between late lactation and early dry period, of which 237 were shared between AL and CTR. The most discriminant compounds (pentosidine and luteolin 7-*O*-glucoside) were related to the more fibrous diet. Pathway analysis indicated that pyrimidine and glycerophospholipid metabolisms were down-accumulated, suggesting reduced rumen microbial activity and liver load. Samples from AL were discriminated from CTR either the day of dry-off or 7 days after. At dry-off, aloin and emodin were the most discriminant metabolites, indicating that *Aloe*'s bioactive compounds were absorbed. Seven days later, 534 compounds were different between groups, and emodin was among the most impacted. Pathway analysis highlighted that glycerophospholipid, pyrimidine, and folate metabolisms were affected. These results might indicate that *Aloe* has positive effects on liver function and a modulatory effect on rumen fermentation.

## Introduction

The dry-off represents a stressful event for dairy cows, encompassing an abrupt milking cessation, diet and group changes, and consequent physiological alterations^1,2^. In fact, the dry-off causes impairments of metabolism, liver function, and antioxidant system, and also triggers an inflammatory response^3–5^. Recently, these changes have been intensively investigated^5–7^, but a lot of work is still required to decipher all the pathways involved. Dry-off is a transitional phase that takes place during the last weeks of gestation and is characterized by biological variations related to the changes in diet and hormonal and metabolic settings^5^. In contrast, the transition period in dairy cows represents the three weeks before and after calving^8,9^ and is characterized by sudden and dramatic changes including alterations in metabolism, inflammatory response, oxidative stress, and a reduction in immune competence^10^. At dry-off, all these phenomena are present^4,6,11^, even though their magnitude is usually lower.

To cope with similar drastic physiological changes to those occurring at dry-off, the administration of nutraceuticals during the transition period has shown positive results in regulating immune responses and metabolism^12^. However, literature addressing the application of these products at dry-off is scarce. *Aloe* spp. are among the most used plants in traditional medicine for their therapeutic properties, such as wound healing, anti-inflammatory, antioxidant, antitumor, antimicrobial, and immunomodulatory effects^13^. The most relevant active compounds of *Aloe* are anthraquinones and their glycosides, which are present in the leaf green rind, and complex carbohydrates, which are detectable in the parenchyma^14^. The biological activities of *Aloe* are likely related to a synergic action of several compounds^14^, but the two main components are aloin, a C-glycoside derivative of a 1,8-dihydroxyanthraquinone, and an acemannan (a typical D-isomer mucopolysaccharide of *Aloe* leaves)^15^. The use of *Aloe arborescens* has been evaluated in dairy cows. Aloin appears to be effectively absorbed, since aloin is detectable in the blood of dairy cows 2 h after oral administration of 200 g/d of whole leaves *A. arborescens* homogenate, without any adverse effect on feed intake and digestibility^16^. Moreover, *A. arborescens* supplementation during transition period showed a positive impact on lipid mobilization, liver and kidney function, and mitigated the inflammatory response at calving^17^. Recently, we tested the use of *A. arborescens* as a lyophilized powder at dry-off^18^. The lyophilization of the homogenate can facilitate the storage, preservation, and administration of the product, which are impractical on commercial farms. Cows that received *A. arborescens* had a slightly different rumen fermentation pattern and improved liver function at dry-off^18^. Moreover, they had a reduced inflammatory response later at calving and higher milk production in the first weeks of lactation. However, the exact mode of action of *A. arborescens* has yet to be fully elucidated.

Omics approaches have been recently used by researchers to gain a better understanding of both cow physiology^19–21^ and different feeding strategies^22,23^. Particularly, metabolomics is a high-throughput technique that allows for the concurrent determination of myriad metabolites in several biological samples^24^, consisting of untargeted and targeted approaches. Targeted metabolomics is used to quantitatively detect metabolites in metabolic pathways of interest, whereas untargeted metabolomics examines metabolite differences between control and experimental groups, which also plays an important role in marker screening for a certain disease or treatment^19^. Of interest, metabolomics has been used to study the transition period^25–27^ and phytogenic compound action in animal models^28^. Moreover, blood metabolomics has been extensively applied in the last few years to identify markers of several diseases^29–32^. However, to the best of our knowledge, studies correlating blood metabolomics to the dry-off period are still lacking.

Therefore, in this study, we used an untargeted metabolomic approach to analyze the plasma metabolite profile. The first aim was to assess whether *A. arborescens* metabolites could be detected in plasma with this technique. Then, according to previous results, we hypothesized that dry-off (performed without antibiotic treatment) would alter the plasma metabolome and lyophilized *A. arborescens* Mill. supplementation could partially improve the physiological response in this phase.

## Results and discussion

To the best of our knowledge, a metabolomic approach has not yet been applied to study the dry-off phase in dairy cows. Nevertheless, both targeted and untargeted metabolomic approaches have been mainly used to explore the periparturient period in dairy cows and the supplementation with different products^25,33^. In particular, untargeted metabolomics can provide new insights into dairy cow physiology in critical phases of the lactation cycle. Therefore, in the present study, we used untargeted metabolomics, based on liquid chromatography coupled with high-resolution mass spectrometry and multivariate statistical modeling, to characterize the biochemical changes occurring in plasma in response to either dry-off or *A. arborescens* supplementation.

### Plasma metabolome profile

**Figure 1:**
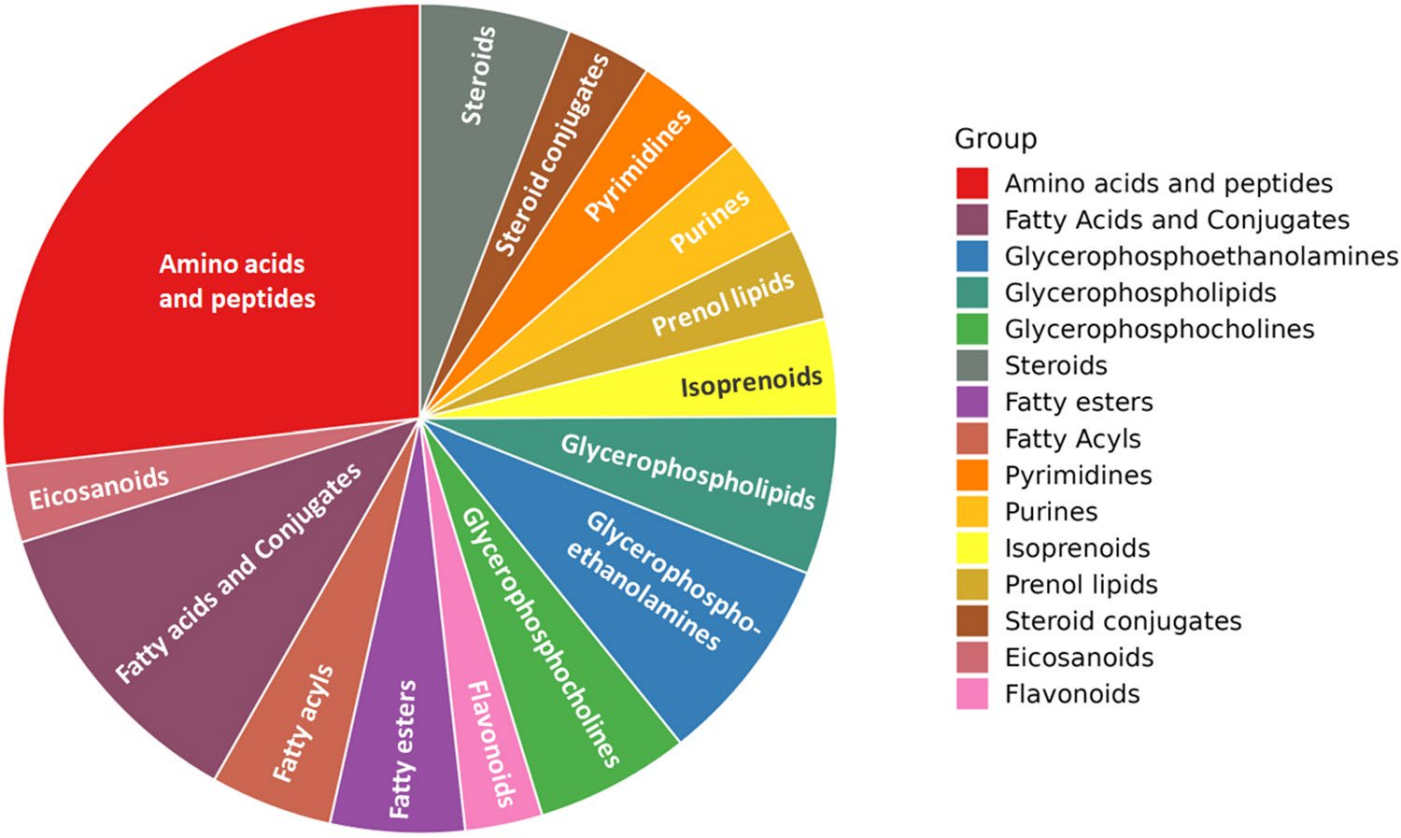
Pie chart showing the chemical classes annotated by untargeted metabolomics in the different plasma samples considering the classes of metabolites containing at least two entries.

The untargeted metabolomic analysis resulted in the identification of 1657 mass features, that were annotated according to a Level 2 of confidence, including both putative annotation and MS/MS confirmation against a comprehensive ad-hoc database of mass spectra (Bovine Metabolome Database^34^). A comprehensive list reporting each annotated metabolite together with its composite mass spectrum, total annotation score, and relative abundance value can be found in Supplementary Table S1. Under our experimental conditions, a wide variety of chemical entities could be recorded, and the most represented classes of compounds detected are reported in Fig. 1. The high number of blood metabolites obtained by the untargeted metabolomic approach denotes the complexity of the biofluid investigated. Overall, amino acids and peptides were by far the most numerically represented class (170 compounds), followed by several sub-classes of lipids and derivatives (such as fatty acids, glycerophosphoethanolamines, glycerophospholipids, glycerophosphocholines, steroids, fatty esters, and fatty acyls). Among the annotated amino acids, we found all those classified as aromatic (i.e. phenylalanine, tryptophan, and tyrosine), together with proline, histidine, lysine, serine, cysteine, glutamine, leucine, methionine, arginine, and glutamic acid (Supplementary Table S1). Of interest, several peptides characterized the dataset, with a great abundance of dipeptides. Also, some metabolites associated with protein mobilization in cattle, such as 3-methylhistidine^35^, creatine, and creatinine were detected. Lipids and lipid metabolism are key aspects of the physiology of transition cows^36,37^ but, to some extent, have been studied also in relation to late lactation and dry-off^38–40^. Additionally, metabolomics allowed the identification of feed-derived compounds (such as isoprenoids and flavonoids), together with purine and pyrimidine derivatives (cumulatively accounting for 53 compounds), prenol lipids (including terpenoids), and some endocrine mediators (i.e. eicosanoids). Among feed-derived compounds, we found hippuric acid. Hippuric acid is formed in the liver to detoxify benzoic acid (a phenolic acid) in the reaction with glycine and can represent an indicator of plant digestion^41^. Furthermore, looking at some nucleic acid intermediates, uric acid was observed in each plasma sample. The latter is produced from the oxidation of xanthine via xanthine dehydrogenase in the intestinal mucosa, liver, and blood^41^. Taken together, the preliminary information obtained by exploring the plasma metabolome profile was coherent with the complex phenomena under investigation, justifying further data reduction and extrapolations to identify the key biomarkers involved.

### Effect of dry-off

**Figure 2:**
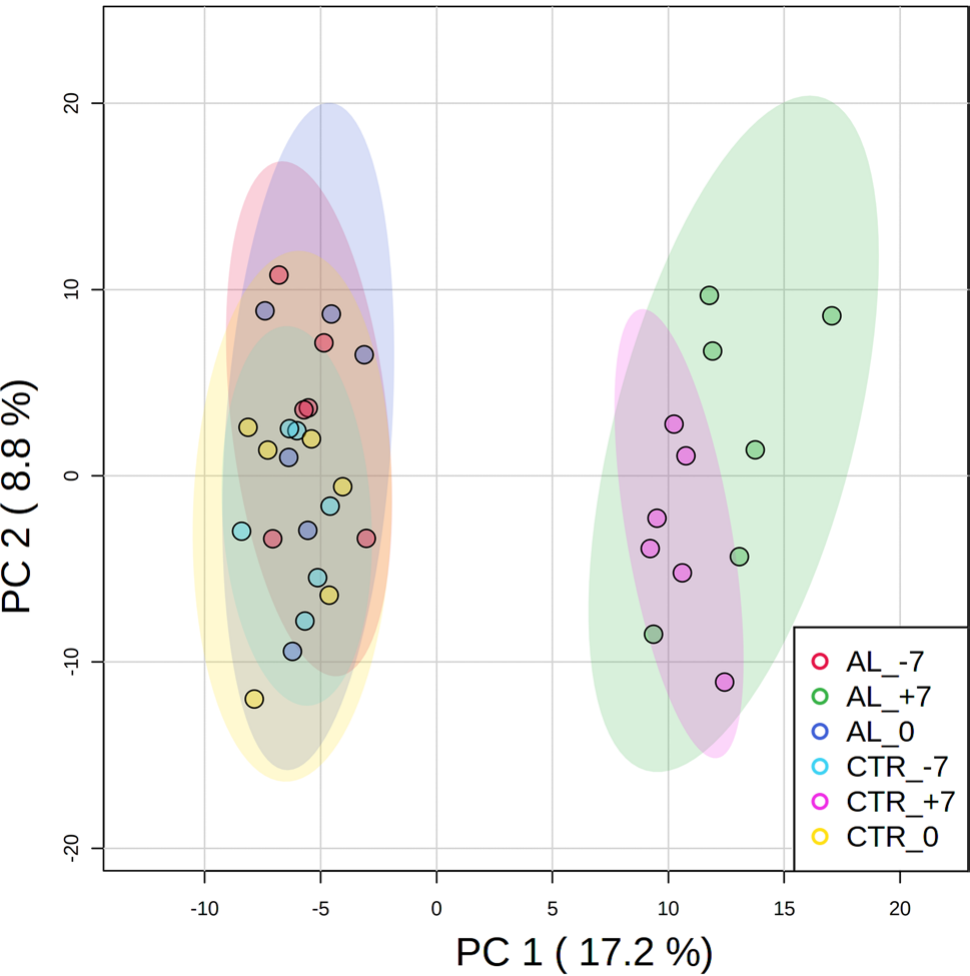
Principal component analysis carried out on plasma samples collected at ‒7, 0, and 7 days from dry-off (DFD) in dairy cows receiving 10 g/d of lyophilized *Aloe arborescens* Mill. (AL) from ‒7 to 7 DFD or in the control group (CTR).

**Figure 3:**
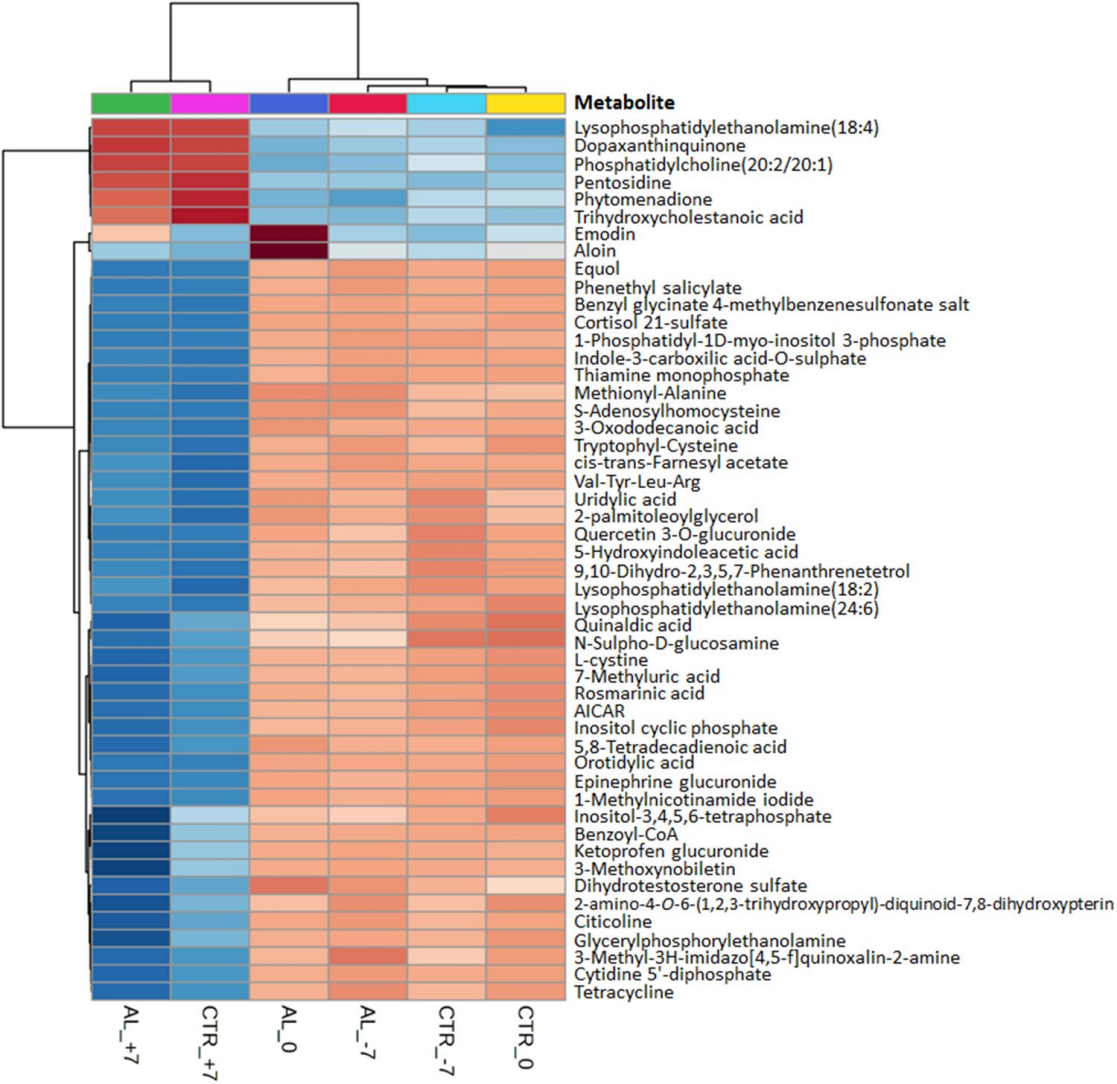
Heatmap with hierarchical cluster analysis showing the longitudinal changes in the plasma metabolome profile across sampling day (‒7, 0, and 7 days from dry-off) and dietary treatment (lyophilized *A. arborescens* supplementation [AL] or control diet [CTR]). Groups at each sampling day are represented in the columns. The top 50 metabolites were ranked by the *P*-value of one-way ANOVA and represented in the lines. Data were auto-scaled by features.

Regardless of dietary treatment, the unsupervised principal components analysis (PCA) discriminated the samples taken at the beginning of dry period from those collected in late lactation (Fig. 2). The first principal component explained around 17% of the total variance, thus suggesting the presence of great variability among samples. The dry-off is a moment of intense physiological and metabolic modifications^5–7^, during which milking is stopped. To firstly depress milk synthesis stimuli and then adapt to the lower demand of the mammary gland, the energy content of the diet is reduced, increasing the fiber fraction. Consistently, the heatmap resulting from the unsupervised hierarchical cluster analysis (Fig. 3) showed a strong clustering of samples taken after dry-off (i.e. 7 DFD). Metabolomic profiles in AL and CTR at 7 DFD clustered together, and they were markedly different from those observed in late lactation (i.e. ‒7 or 0 DFD). Results obtained with this omics approach confirmed previous observations made with other techniques highlighting a different overall metabolic state between lactating and nonlactating periods.

To identify the metabolites that were mostly affected by the dry-off event, supervised OPLS-DA models were applied separately to AL and CTR groups, comparing samples before dry-off with those collected after milking cessation (7 DFD; Supplementary Fig. S1). This technique allows us to identify the most discriminant compounds (i.e. the plasma metabolites driving the discrimination according to the cluster type) between lactation and early dry period. OPLS-DA is widely used in metabolomic research for identifying those biomarker compounds maximizing group separation^23^. In both treatment groups, the score plots separated samples taken before dry-off from those collected in early dry period, confirming the results of the unsupervised PCA. This outcome was also supported by the validation parameters, being the goodness-of-fit (R^2^Y) = 0.99 and the goodness-of-prediction (Q^2^) = 0.92. The discriminant compounds were extrapolated by VIP selection method (Table 1), and 490 compounds showed the highest prediction ability (VIP score ≥ 1). Among those, 127 were exclusively differentially expressed in AL, 126 only in CTR, and 237 were shared between the groups. Looking at the common discriminant compounds, the most represented and enriched classes were amino acids and peptides (20 compounds, with a great abundance of dipeptides), steroids (9 compounds, including some metabolites of progesterone and 17-β-estradiol), pyrimidines (8 compounds, including orotidylic acid, thiamine, and dihydrouracil), glycerophosphoethanolamines (7 compounds), and indoles (including some tryptophan derivatives, such as 5-methoxytryptophan and tryptophanol). Interestingly, among the most discriminant compounds in both models pentosidine and luteolin 7-*O*-glucoside were present. The first one is an advanced glycation end product, linked to the Maillard reaction. The up-accumulation of this compound after dry-off might be related to dietary intake, due to Maillard reaction happening during the sun drying of the hay fed^42^. However, it is usually rapidly excreted by glomerular filtration in healthy subjects^43^. Thus, alongside the latter, another possible explanation could be represented by a transient impairment of kidney function in this phase. The latter could be supported by the higher plasma concentration of creatinine after dry-off^5,44^, since circulating creatinine is related to the kidneys’ ability to remove it from the bloodstream^45^. Moreover, advanced glycation end products are recognized as pro-inflammatory mediators, their formation is related to that of reactive oxygen metabolites, and can be formed as a result of oxidative stress and along inflammatory pathways^46^. Oxidative stress and inflammation can happen as a result of dry-off^5^. Luteolin 7-*O*-glucoside, instead, is found in grass hay^47^. Its increase was related to the hay-based diet fed after dry-off, which drastically changed from the lactation diet, which had lower hay inclusion and higher amount of silages and concentrates.

**Table 1:** Most discriminant compounds (according to variable importance in projection [VIP]) between plasma samples collected before (‒7 and 0 days from dry-off [DFD]) or after dry-off (7 DFD).

Compound	VIP score	cvSE^a^	LogFC^b^
Citicoline	4.78	0.73	− 9.33
Epinephrine glucuronide	4.51	1.27	− 8.81
Benzoyl-CoA	4.39	0.93	− 8.27
Dopaxanthin quinone	4.37	1.09	9.09
Pentosidine	4.29	1.26	8.62
Phosphatidylinositol 3-phosphate	4.29	1.88	− 8.03
Tetracycline	4.05	0.87	− 7.16
Indole-3-carboxylate-*O*-sulphate	3.94	0.88	− 6.48
S-Adenosylhomocysteine	3.92	1.34	− 6.77
Benzyl glycinate 4-methylbenzenesulfonate	3.80	0.70	− 5.88
Phytomenadione	3.72	1.11	6.17
Luteolin 7-*O*-glucoside	3.70	1.57	6.75
Quetiapine	3.56	1.97	− 5.76
Lysophosphatidylethanolamine(18:4)	3.47	1.22	5.79
Maltohexaose	3.44	2.50	6.20
(3α,7 α,12 α)-Trihydroxy-5β-cholestanoic acid	3.43	1.29	5.40
Cytidine 5'-diphosphate	3.37	0.97	− 4.98
Triacylglycerol(18:3/22:6/22:2)	3.36	1.32	5.48
6-Succinoaminopurine	3.34	1.15	6.05
Tryptophyl-cysteine	3.18	1.25	− 4.20

^a^cvSE, cross-validation standard error.

^b^LogFC, logarithm of Fold-Change values.

From the pathway analysis conducted on each group comparing samples before dry-off with those collected at 7 DFD, there were 2 shared pathways. The most important was the pyrimidine metabolism pathway (*P* < 0.01). Pyrimidine metabolites were mostly down-accumulated, likely indicating a diminished rumen microbial N production. Since pyrimidines are nucleic acid constituents and the amount of DNA and RNA synthesized in the rumen depends largely on bacterial growth^48,49^, we hypothesized that the reduced rumen fermentation intensity after dry-off^50^ could account for this result.

Liver function is transitorily impaired after dry-off^5^. In the present study, this was supported also by the down-accumulation of compounds taking part in the glycerophospholipid metabolism pathway (*P* < 0.01). Glycerophospholipids are involved in triglyceride export by the liver, playing a key role in very low-density lipoprotein (VLDL) hepatic synthesis. Therefore, the mild increase in plasma nonesterified fatty acids (NEFA) usually observed immediately after dry-off^5,6,18^ might be interpreted as a decreased liver ability to remove NEFA from the bloodstream rather than excessive fat mobilization. Glycerophospholipid down-accumulation after dry-off might also be linked to a reduced liver load driven by the lower intake, which makes less necessary VLDL synthesis.

### Effect of Aloe supplementation

Previous research showed the beneficial effects of *A. arborescens* supplementation during transition^17^ and dry-off periods^18^. Particularly, *A. arborescens* homogenate supplementation during transition period resulted in lower plasma concentrations of NEFA, β-hydroxybutyrate (as markers of reduced lipid mobilization), myeloperoxidase, and ceruloplasmin, and higher concentrations of cholesterol, retinol, tocopherol, and paraoxonase (as markers of mitigated inflammatory response at calving), also paired with lower plasma concentrations of creatinine and bilirubin (indicating an improved kidney and liver function)^17^. At dry-off (in the same cows of this study), lyophilized *A. arborescens* supplementation led to higher plasma concentrations of paraoxonase and glucose, and lower urea^18^, suggesting a better liver function. Paraoxonase is a liver enzyme that is released into the bloodstream and its decrease can be interpreted as a liver impairment indicator^51^. Similarly, glucose supply heavily relies on liver endogenous production, particularly during transition period^52^.

**Figure 4:**
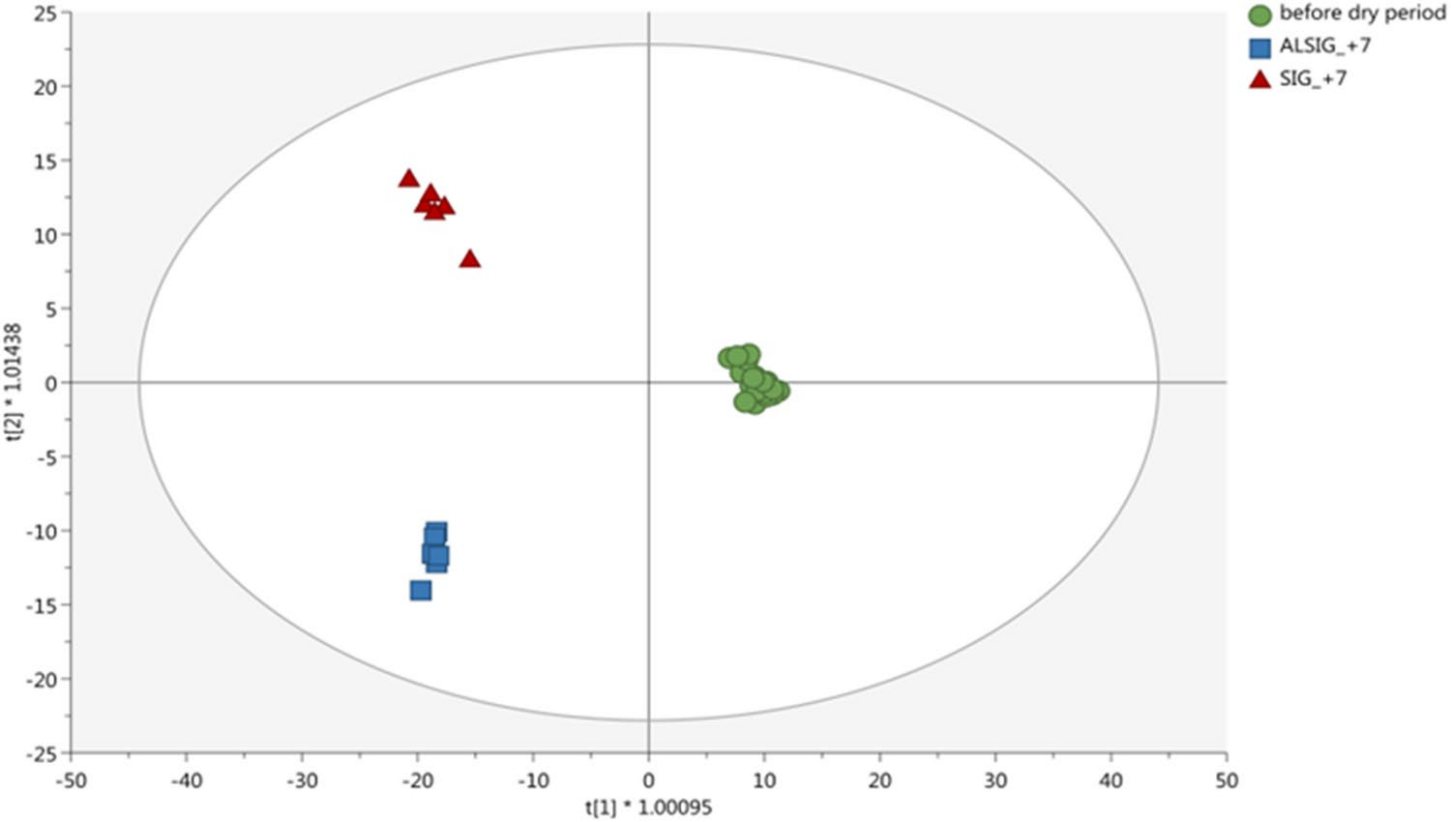
Orthogonal projection to latent structures (OPLS) discriminant analysis (DA) considering the cluster type as class discrimination parameter. Plasma samples were collected at ‒7, 0, and 7 days from dry-off (DFD) in dairy cows receiving 10 g/d of lyophilized *Aloe arborescens* Mill. (AL) from ‒7 to 7 days relative to dry-off or in the control group (CTR). Samples taken before dry-off (‒7 and 0 DFD) were considered as a reference. The comparison between samples collected at 7 DFD in AL and CTR was further discussed.

Consistently with those results, alongside the sampling day effect, the heatmap reported in Fig. 3 also shows the clustering of samples according to the *Aloe* treatment. There was a clear grouping between AL and CTR at 7 DFD, and samples taken at 0 DFD in AL were markedly different from their counterparts in CTR, with aloin and emodin being the most discriminant compounds. In our previous paper^18^, we showed positive effects of *A. arborescens* supplementation. The annotation of these compounds in the plasma of supplemented cows biologically supported those results showing that *Aloe* bioactive compounds were effectively absorbed. Emodin is a natural anthraquinone derivative with a wide spectrum of pharmacological properties, including anticancer, hepatoprotective, anti-inflammatory, antioxidant, and antimicrobial activities^53^ and it represents, together with aloin, one of the main secondary phenolic metabolites of *Aloe*^54^. It takes its origin from the conversion of barbaloin (a mixture of two diastereomers, aloin A and aloin B) by intestinal bacteria. Previous research suggested that aloin can be metabolized into aloe-emodin in the intestine and then absorbed into the bloodstream^55^. However, Bani et al.^16^ successfully detected aloin, but not emodin, in plasma of dairy cows supplemented with 200 g/d of *A. arborescens* homogenate. This discrepancy between these two studies could be related to the different analytic techniques used, mainly in terms of FWHM resolution (liquid chromatography coupled to triple quadrupole mass spectrometry *vs* ultra-high-pressure liquid chromatography coupled with orbitrap mass spectrometry), or to the different forms of supplementation (lyophilized *vs* homogenate). High mass resolution is particularly important for all types of experiments involving complex mixtures, such as samples generated from a biological matrix (such as plasma, milk, and others), since these latter potentially contain a significant number of background (matrix) ions in addition to the possible analytes of interest. In such cases, high mass resolution makes the difference between detecting analyte molecules at low concentration and not detecting them due to the masking effect of isobaric matrix interferences^56,57^. Then, considering that samples taken at 7 DFD were different from those before dry-off and that a tendency toward separation between AL and CTR was observed at 7 DFD (Supplementary Fig. S1), we focused our analysis on the early dry period when, also in our previous paper^18^, the largest differences were noted. Another OPLS-DA model was applied to the data, considering three clusters: samples taken before dry-off and samples taken at 7 DFD either in AL or CTR (Fig. 4). The plot showed a clear separation between the clusters (R^2^Y = 0.98 and Q^2^ = 0.55). As far as the VIP scores are concerned, 534 compounds showed the highest prediction ability (VIP score ≥ 1) thus successfully discriminating AL from CTR. Amino acids and peptides were the most represented compounds (54 metabolites), followed by fatty acids and conjugates (21 metabolites), pyrimidines, steroids conjugates, and fatty acyls (13 metabolites each), and isoprenoids, steroids, glycerophospholipids, and glycerophosphoethanolamines (12 metabolites each).

Of interest, the most discriminating compound according to the VIP algorithm between AL and CTR at 7 DFD was emodin (Table 2), consistent with the previous discussion. In this case, aloin was not among those compounds with the highest prediction ability. The reason might be related to the different rumen environment which could have resulted in higher degradation of aloin or a higher conversion into emodin, the latter potentially involving a higher deglycosylation activity at the intestinal level by hydrolyzing enzymes. It is also important to highlight that, in this work, the database used for the annotation does not contain structural information regarding glucuronides/sulfates forms of anthraquinones, which are reported to play an important role in aloin/emodin absorption following the activity of phase II enzymes^58^. Therefore, up to date, limited information is available on anthraquinone bioavailability, and further studies are required to confirm our findings. The second most discriminant ability was outlined for glycerylphosphorylethanolamine. It is a membrane breakdown product resulting from the cleavage of the lipid group from glycerophosphoethanolamine fatty acids (i.e. phosphatidylethanolamine). Its lower relative accumulation in AL (logFC = ‒0.95) was likely related to the aforementioned better liver function observed in those cows, as supported by the higher plasma paraoxonase concentration^18^.

**Table 2:** Most discriminant compounds (according to variable importance in projection [VIP]) between plasma samples collected 7 days from dry-off (DFD) in dairy cows receiving 10 g/d of lyophilized *Aloe arborescens* Mill. (AL) considering ‒7 to 7 days from dry-off or in the control group (CTR).

Metabolite	VIP score	cvSE^a^	LogFC^b^
Emodin	2.32	0.81	3.25
Glycerylphosphorylethanolamine	2.27	0.95	− 0.95
3-Methoxynobiletin	2.26	0.93	− 0.93
Ketoprofen glucuronide	2.26	0.93	− 0.93
Succinylaminoimidazole carboxamide ribotide	2.24	0.97	− 0.95
Geranylgeranylcysteine	2.07	0.66	1.73
4-t-octylphenol	2.06	1.16	− 1.02
Cinnamyl alcohol	2.02	0.76	2.98
Cytidine-diphosphate diacylglycerol (18:0/22:3)	2.02	1.23	0.32
Ethenodeoxyadenosine	2.00	1.01	− 1.09
Sinapic acid	1.99	1.17	0.22
Quinaldic acid	1.99	1.07	− 0.22
Flavin Mononucleotide	1.99	1.08	− 1.01
L-isoleucyl-L-proline	1.98	1.16	− 0.30
D-Xylono-1,5-lactone	1.96	0.96	− 0.79
D-Myo-inositol 3,4,5,6-tetrakisphosphate	1.96	1.37	− 1.82
Triacylglycerol(20:3n6/15:0/O-18:0)	1.91	1.34	0.29
Alpha-N-Phenylacetyl-L-glutamine	1.90	0.90	− 0.62
Vanillylmandelic acid	1.88	1.06	− 1.06
Tricosanoic acid	1.85	1.30	− 0.27

^a^cvSE, cross-validation standard error.

^b^LogFC, logarithm of Fold-Change values.

The compound with the highest fold change was a compound of the class of phosphatidylcholines (logFC = 5.57). In cattle, a decrease in the abundance of lipids related to phosphatidylcholine metabolism was associated with increased severity of fatty liver^59^. Phosphatidylcholine is involved in VLDL synthesis and helps prevent fatty liver, exporting triglycerides from the hepatocytes as VLDL^60,61^. This might help explain the anti-hyperlipidemic role of *Aloe* previously reported^17^ and the improved liver function that we observed^18^.

Then, we investigated the differentially expressed pathways (Table 3). Among those that resulted significant, glycerophospholipid, pyrimidine, folate, nicotinate, and nicotinamide metabolisms were the most impacted. Together, these pathways suggest that liver and rumen metabolism might have been key regulators of the *Aloe* effects described previously^17,18^. Relative plasma concentration of compounds taking part in the glycerophospholipid metabolism (mostly phosphatidylethanolamine and phosphatidylcholine) was reduced. To the best of our knowledge, information about glycerophospholipid metabolism at dry-off is lacking. Nevertheless, it has been reported that, during transition period, blood glycerophospholipid decrease can be interpreted as a marker of fatty liver^36^ but was also associated with a desired healthy metabolic phenotype^25^. In the present experiment, cows supplemented with *A. arborescens* at dry-off seemed to have improved liver function. Thus, the down-accumulation of glycerophospholipids might result from reduced liver load in a phase when the liver has to cope with several adaptations^5^. Alternatively, glycerophospholipid down-accumulation might have been driven by the lower availability of methyl donors, discussed thereafter,

**Table 3:** Significantly impacted pathways by *Aloe* supplementation (AL; 10 g/d of lyophilized *A. arborescens* Mill. from ‒7 to 7 days from dry-off [DFD]) compared with control diet (CTR) in dairy cows at 7 DFD.

Pathway name	Metabolites impacted^a^	*P*-value	Impact
Glycerophospholipid metabolism	8/36	< 0.01	0.52
Pyrimidine metabolism	8/38	< 0.01	0.24
One carbon pool by folate	3/9	0.02	0.91
Purine metabolism	9/66	0.02	0.20
Nicotinate and nicotinamide metabolism	8/13	0.05	0.43

^a^Number of metabolites impacted by *Aloe* supplementation out of the total number of metabolites in the considered pathway.

Pyrimidine relative concentrations were mostly reduced in AL cows compared with CTR. As mentioned before, pyrimidines in the bloodstream are mainly of rumen origin, being constituents of microbial DNA and RNA^23,48,49^. Despite the lack of data after dry-off, the day of dry-off rumen fluid volatile fatty acid concentration tended to be lower in AL cows^18^. Together, these results can indicate that *Aloe* has a modulatory effect on ruminal fermentation. Although in vitro trials did not show any adverse effect of *Aloe* incubation on rumen fermentation parameters^16,62^, in vivo results might be different. We previously hypothesized that a different epithelial absorption rate might also be implied, as suggested by the higher daily rumination time observed^18^. Looking at changes in pyrimidines, we would support the modulatory effect hypothesis rather than the increased absorption, but further studies are needed to disentangle this matter. It may also be hypothesized that *Aloe* could reduce microbial turnover through some effect on protozoa^63^.

Finally, folate metabolites were mostly down-accumulated. Folate cycle is part of the one-carbon metabolism^12,64^. The decrease in plasma concentrations of these compounds might suggest an increased consumption of methyl donors in those cows^60,65^. Therefore, it may be possible to speculate that *Aloe* action was related to some effect on DNA methylation pattern, supporting the mitigated inflammatory response observed two months later at calving^18^. These hypotheses agree with previous findings associating cow condition at dry-off and calving^66^ and might help explain why priming dairy cows long before calving has beneficial effects^9^.

## Conclusions

The dry-off confirmed to be a phase of intense modifications in the metabolism of dairy cows, with deep alterations in metabolism and liver function. Of interest, metabolic pathways involved in pyrimidines and glycerophospholipid metabolisms were impacted by the transition to dry period. At the same time, supplementing lyophilized *A. arborescens* seemed to improve the adaptation to dry period at two levels: at the rumen level and tuning some metabolic pathways. In this study, using an untargeted metabolomic approach, we demonstrated that aloin, the main *Aloe* bioactive compound, is absorbed by cows and metabolized to emodin, supporting previous observations and confirming the positive effect of *Aloe* supplementation on liver function in this phase.

## Methods

### Animal management and experimental design

The research was carried out at the Università Cattolica del Sacro Cuore dairy barn (Cerzoo, San Bonico, Piacenza, Italy) in accordance with animal welfare guidelines (including ARRIVE guidelines) and Italian laws on animal experimentation and ethics (Italian Health Ministry Authorization N 444/2019-PR in agreement with D. Lgs. n. 26, 04/03/2014). This work was part of a larger study and detailed information about animals and experimental design, as well as results in terms of performance, milk, rumen fluid, and plasma analyses have been reported elsewhere^18^. Briefly, 23 Holstein dairy cows with milk somatic cell count less than 200,000 cells/mL and without intramammary infections due to major pathogens (i.e. *Staphylococcus aureus*, *Streptococcus uberis*, Enterobacter spp., *Escherichia coli*, *Trueperella pyogenes*, *Serratia marcescens*) were included in this study. During lactation, cows were housed in a free-stall pen, received lactation diet, and were milked twice daily (5:00 and 17:00 h). About 55 days before expected calving, cows were abruptly dried off with the infusion of internal teat sealant (Noroseal, Norbrook Laboratories Limited, UK) after the last milking. Then, they were moved to a dry pen and received ad libitum grass hay only for 7 days, after which dry period diet was administered. Cows received 10 g/d of lyophilized *A. arborescens* Mill. whole leaf homogenate supplemented to their diet from –7 to 7 days from dry-off (DFD) (AL; n = 11) or none for the control group (CTR; n = 12). Groups were balanced for parity, previous lactation length, and somatic cell history. Before total mixed ration (TMR) distribution, feed bunk was cleaned, all cows were restrained in the headlocks, and each dose of lyophilized *Aloe* was mixed with 1 kg of lactation TMR and fed to AL cows, whereas CTR cows received 1 kg of lactation TMR only. During the whole trial, an operator checked that cows ate all the supplemented TMR.

### Blood samples

Blood samples were collected from the jugular vein into heparinized tubes before the morning feeding on –7, 0, and 7 DFD. Tubes were immediately cooled in an ice-water bath. Afterwards, whole blood was centrifuged at 3500 × g for 15 min at 4 °C, and plasma was stored at –20 °C for subsequent assays. A subset of 6 cows/group was randomly selected and samples from these cows underwent metabolomic analysis, as described below.

The extraction of metabolites from plasma samples was carried out as previously reported by Luo et al.^27^, with few modifications. Briefly, seventy-two plasma samples were slowly thawed at 4 °C, and from each sample, a 200-μL aliquot was taken and added to 800 μL of pre-cooled methanol/acetonitrile solution (1:1, vol/vol). Samples were then vortex mixed and maintained at ‒20 °C for 60 min, followed by centrifugation at 14,000×*g* and 4 °C for 20 min. The resulting supernatants were finally filtered through 0.22-μm cellulose syringe filters in ultra-high-pressure liquid chromatography (UHPLC) vials until further untargeted metabolomic profiling.

### Untargeted metabolomic analysis

The untargeted UHPLC-HRMS analysis was done using a Q Exactive™ Focus Hybrid Quadrupole-Orbitrap Mass Spectrometer (Thermo Scientific, Waltham, MA, USA) coupled to a Vanquish UHPLC pump, equipped with heated electrospray ionization (HESI)-II probe (Thermo Scientific, USA). A water-acetonitrile (both LC–MS grade, from Sigma-Aldrich, Milan, Italy) gradient elution (6–94% acetonitrile in 35 min) was done for the chromatographic separation, using 0.1% formic acid as phase modifier, exploiting an Agilent Zorbax Eclipse Plus C18 column (50 × 2.1 mm, 1.8 μm). The flow rate was 200 μL/min, considering an injection volume of 6 μL. The untargeted analysis consisted of a full scan in the 100–1200 m/z range, using a positive ionization mode and setting a mass resolution of 70,000 at m/z 200. Automatic gain control (AGC) target and maximum injection time were 1 × 10^6^ and 200 ms, respectively. Under our instrumental conditions, pooled quality control samples were randomly injected and analyzed in a data-dependent (Top N = 3, under a stepped normalized collisional energy) MS/MS mode, with full scan mass resolution reduced to 17,500 at m/z 200, AGC target of 1 × 10^5^, maximum injection time of 100 ms, and isolation window of 1.0 m/z. The HESI parameters are reported elsewhere^23^. The mass spectrometer was calibrated using Pierce positive ion calibration solution (Thermo Fisher Scientific, San Jose, CA, USA) before the UHPLC-HRMS run.

The collected raw data were further processed using the software MS-DIAL (version 4.70) for the automatic peak finding, LOWESS normalization, and annotation via spectral matching against the comprehensive Bovine Metabolome Database^34^ (last accessed date: 15/11/2022). The MS and MS/MS tolerance for peak centroiding was set to 0.01 and 0.05 Da, respectively, considering features with a minimum peak height of 10,000 cps^23^. Therefore, the identification step was based on mass accuracy, isotopic pattern, and spectral matching. The selected identification criteria were used to calculate a total identification score. The total identification score cut-off was > 50%, considering the most common source adducts. Additionally, a gap filling (using the peak finder algorithm) was done to fill in missing peaks, setting a 5-ppm tolerance for m/z values.

### Statistical analysis

The metabolomics-based dataset was further elaborated for multivariate statistical modeling using two different software, namely MetaboAnalyst^67^ and SIMCA 13 (Umetrics, Malmo, Sweden)^23^. Data were log-transformed, and Pareto scaled. After normalization, both unsupervised and supervised multivariate statistics were carried out. The unsupervised approach was based on both PCA and hierarchical cluster analysis, while the orthogonal projections to latent structures discriminant analysis (OPLS-DA) was considered as a supervised tool. The OPLS-DA model validation parameters (goodness-of-fit R^2^Y together with goodness-of-prediction Q^2^Y) were recorded, considering a Q^2^Y prediction ability of > 0.5 as the acceptability threshold^23^, and excluding model overfitting by permutation testing (N > 100). The discriminant potential of each plasma metabolite was then calculated according to the variable selection method variable importance in projection (VIP), using as the minimum significant threshold a VIP score ≥ 1. Finally, the software MetaboAnalyst was used initially to inspect those metabolic pathways mostly represented by the metabolites annotated (using as pathway library: *Bos taurus*, KEGG), and then to provide a metabolite set enrichment analysis based on those significant classes of metabolites/metabolic pathways revealed by multivariate statistics.

### Ethics declaration

The study was approved by the Università Cattolica del Sacro Cuore Animal Welfare Committee (OPBA) and authorized by the Italian Health Ministry (Authorization N 444/2019-PR in agreement with D. Lgs. n. 26, 04/03/2014).

## Supplementary Information

Supplementary Figures.

Supplementary Table S1.

## Data Availability

The dataset used and analyzed during the current study is available in Supplementary Table S1.
