# Supplementary material for: Impact of dry-off and lyophilized Aloe arborescens supplementation on plasma metabolome of dairy cows
Source: Sci Rep. 2023 Mar 31;13:5256. doi: 10.1038/s41598-023-31922-z (PMC10066363; doi:10.1038/s41598-023-31922-z)
Supplement: Supplementary file 1 — Supplementary Figures. [file 41598_2023_31922_MOESM1_ESM.docx]

**Impact of dry-off and lyophilized *Aloe arborescens* supplementation on plasma metabolome of dairy cows.** L. Cattaneo, G. Rocchetti, F. Piccioli-Cappelli, S. Zini, E. Trevisi, and A. Minuti

**
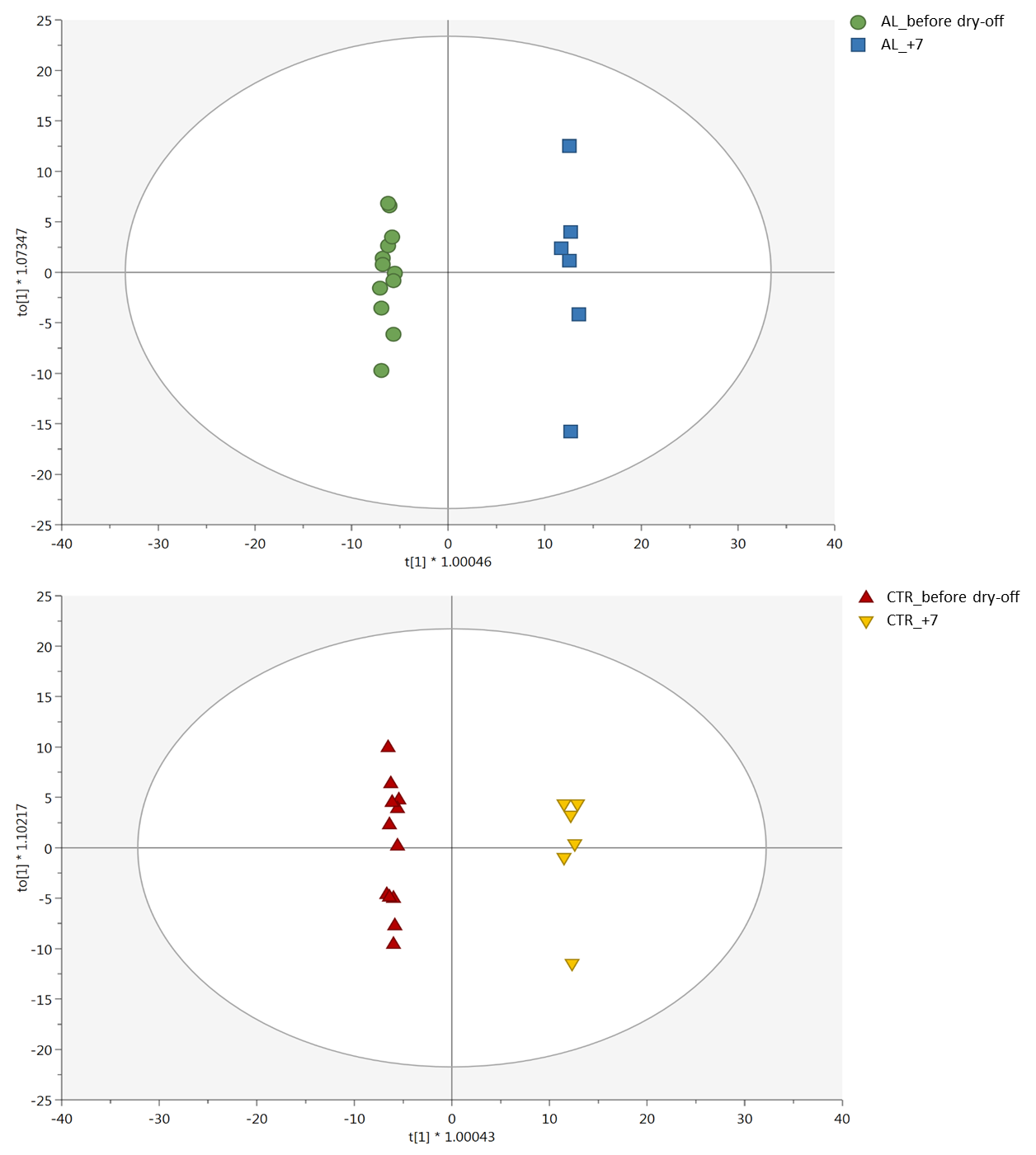
**

**Supplementary Figure S1**. Orthogonal projection to latent structures (OPLS) discriminant analysis (DA) considering the cluster type as class discrimination parameter. Plasma samples were collected at ‒7, 0, and 7 days from dry-off (DFD) in dairy cows receiving 10 g/d of lyophilized *Aloe arborescens* Mill. (AL) from ‒7 to 7 days relative to dry-off or in the control group (CTR). In each treatment group, samples taken before dry-off (‒7 and 0 DFD) were compared with those collected at 7 DFD.


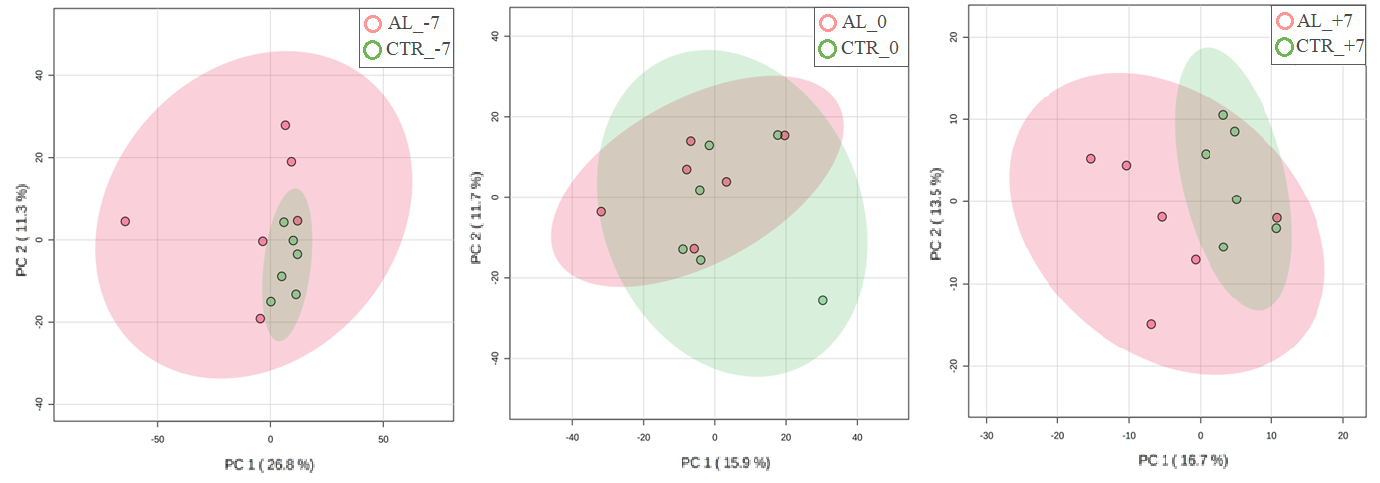


**Supplementary Figure S2**. Principal component analysis carried out separately on the three sampling days on plasma samples collected at ‒7, 0, and 7 days from dry-off (DFD) in dairy cows receiving 10 g/d of lyophilized *Aloe arborescens* Mill. (AL) from ‒7 to 7 DFD or in the control group (CTR).
